# Cell-Type Dependent Regulation of the Electrogenic Na^+^/HCO_3_^−^ Cotransporter 1 (NBCe1) by Hypoxia and Acidosis in Glioblastoma

**DOI:** 10.3390/ijms23168975

**Published:** 2022-08-11

**Authors:** Marina Giannaki, Debora E. Ruf, Emilie Pfeifer, Katharina Everaerts, Dieter H. Heiland, Oliver Schnell, Christine R. Rose, Eleni Roussa

**Affiliations:** 1 Department of Molecular Embryology, Faculty of Medicine, Institute of Anatomy and Cell Biology, Albert-Ludwigs-Universität Freiburg, Albertstrasse 17, D-79104 Freiburg, Germany; 2 Institute of Neurobiology, Heinrich Heine University, D-40225 Düsseldorf, Germany; 3 Department of Neurosurgery, Faculty of Medicine, Medical Center, University of Freiburg, D-79106 Freiburg, Germany; 4 Microenvironment and Immunology Research Laboratory, Medical Center, University of Freiburg, D-79106 Freiburg, Germany

**Keywords:** pH regulation, growth factors, cancer

## Abstract

Glioblastoma multiforme (GBM) is the most common and malignant brain tumour. It is characterised by transcriptionally distinct cell populations. In tumour cells, physiological pH gradients between the intracellular and extracellular compartments are reversed, compared to non-cancer cells. Intracellular pH in tumour cells is alkaline, whereas extracellular pH is acidic. Consequently, the function and/or expression of pH regulating transporters might be altered. Here, we investigated protein expression and regulation of the electrogenic sodium/bicarbonate cotransporter 1 (NBCe1) in mesenchymal (MES)-like hypoxia-dependent and -independent cells, as well as in astrocyte-like glioblastoma cells following chemical hypoxia, acidosis and elucidated putative underlying molecular pathways. Immunoblotting, immunocytochemistry, and intracellular pH recording with the H^+^-sensitive dye 2′,7′-bis-(carboxyethyl)-5-(and-6)-carboxyfluorescein were applied. The results show NBCe1 protein abundance and active NBCe1 transport. Hypoxia upregulated NBCe1 protein and activity in MES-like hypoxia-dependent GBM cells. This effect was positively correlated with HIF-1α protein levels, was mediated by TGF-β signalling, and was prevented by extracellular acidosis. In MES-like hypoxia-*in*dependent GBM cells, acidosis (but not hypoxia) regulated NBCe1 activity in an HIF-1α-independent manner. These results demonstrate a cell-specific adaptation of NBCe1 expression and activity to the microenvironment challenge of hypoxia and acidosis that depends on their transcriptional signature in GBM.

## 1. Introduction

pH regulatory systems in tumour cells are targets for approaches in cancer therapy. In tumours, altered cell metabolism and the subsequent adaptation of acid/base machineries lead to a reversal of pH gradients between the intracellular and extracellular compartments compared to healthy tissue. In tumour cells, intracellular pH (pH_i_) is mildly alkaline, favouring cell proliferation, whereas the extracellular pH is acidic, favouring tumour metastasis [1,2,3]. Tumour microenvironment is additionally characterised by hypoxia, due to impaired vascularisation. Thus, in order to be able to survive and proliferate, tumour cells have to cope with and adapt to two different extracellular pathological challenges, i.e., hypoxia and acidosis. Thereby, upregulation of hypoxia inducible factors (HIFs) is a key molecular event, followed by subsequent altered expression of many genes and proteins [4,5]. Among acid/base transport proteins, regulation of Na^+^/H^+^ exchanger 1 (NHE1), the major pH_i_ regulator in cancer cells, Na^+^/HCO_3_^−^ cotransporters (NBCs), anion exchangers (AEs), subunits of vacuolar H^+^-ATPase (ATP6V1), together with carbonic anhydrase (CA) and monocarboxylate transporter 4 (MCT4) have been investigated in the context of hypoxia and/or acidosis in cell lines derived from different cancer types [2].

Glioblastoma multiforme (GBM) is the most common and malignant brain tumour in adults, with a median survival period of 13 months [6]. Treatment failure and tumour relapse have been linked to glioblastoma hallmarks, such as an intratumoral cellular heterogeneity [7,8,9]. Based on transcriptional profiles, malignant cells in glioblastoma are classified into four main cellular subtypes, namely neural progenitor-like (NPC-like), oligodendrocyte-progenitor-like (OPC-like), astrocyte-like (AC-like) and mesenchymal-like (MES-like) cells [8,10]. These subtypes occur simultaneously within the same tumour, thus having broad implications for therapy [9,10]. Additionally, the MES-like subtype has been further categorized into hypoxia-dependent and a hypoxia-*in*dependent subgroups based on the strong association with hypoxia-response-, stress-, and glycolytic genes expression [10]. The most recent classification of the spatial architecture of glioblastoma confirmed that the hypoxia-associated gene expression subgroup occupied segregated niches within the tumour highly associated with genomic instability and tumour resistance [11].

*Slc4a4,* the gene that encodes NBCe1, is regulated by hypoxia in several epithelial cancer cell lines, but not in glioblastoma cell lines [12]. In quiescent glioblastoma cancer stem-like cells maintained in an acidic environment, WNK1-dependent NBC phosphorylation has been proposed to be linked to their response to treatment approaches [13]. However, the adaptation of pH regulatory proteins (specifically NBCs) in well-characterised tumour cell subpopulations, to changes in the tumour microenvironment has not yet been addressed. In the present study, we sought to investigate the expression and regulation of NBCe1 in MES-like hypoxia-dependent and *in*dependent cells, as well as in AC-like glioblastoma cells following chemical hypoxia alone or in combination with extracellular acidosis and have elucidated putative underlying molecular pathways.

## 2. Results

### 2.1. NBCe1 Protein Abundance and Activity in Glioblastoma Cellular Subtypes

**Figure 1:**
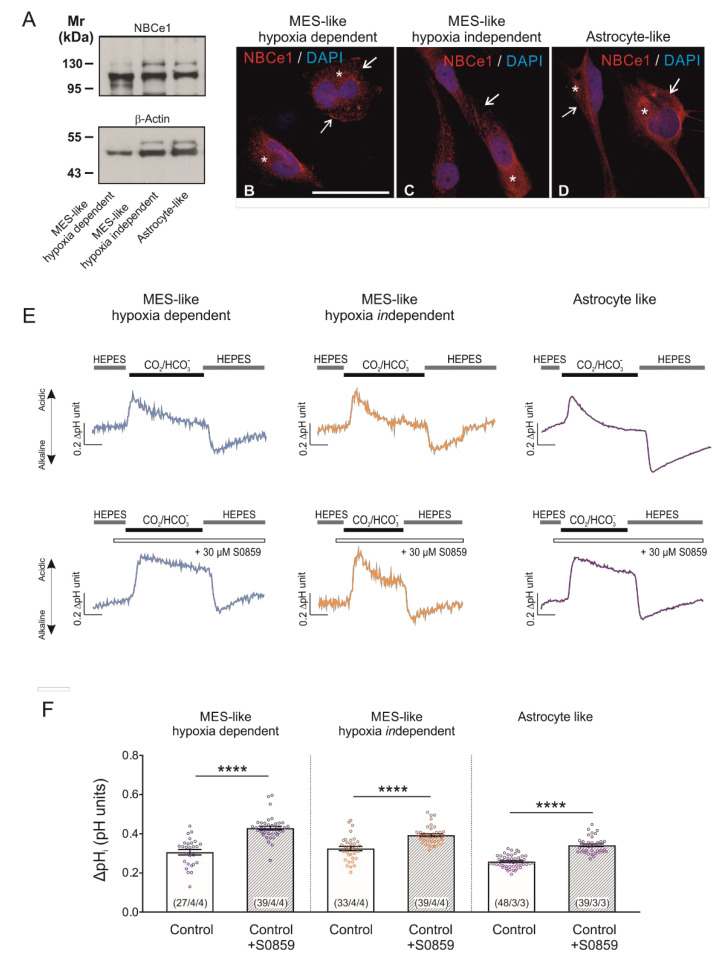
Baseline protein expression of NBCe1 and active NBC transport in glioblastoma cellular types. (**A**) Protein abundance of NBCe1 by immunoblotting in mesenchymal-(MES)-like hypoxia-dependent, MES-like hypoxia-*in*dependent and astrocyte-like glioblastoma cells; 10 µg protein was loaded per lane. The blots are representative of three different experiments. (**B**–**D**) Immunofluorescence for NBCe1 (red) and subsequent confocal microscopy in different glioblastoma cellular subtypes. Arrows point to membrane NBCe1 and asterisks indicate intracellular NBCe1 distribution. Nuclear staining with DAPI. Scale bar: 50 µm. (**E**) Analysis of NBC transport activity in MES-like hypoxia-dependent and hypoxia-*in*dependent cells, as well as in astrocyte-like glioblastoma cellular subtypes. Original recordings of intracellular pH in cultured GBM cells during change of perfusion solution from HEPES-buffered to saline solution buffered by 5% CO_2_/26 mM HCO_3_^-^ and back to HEPES in the presence or absence of 30 µM of the NBC inhibitor S0859. (**F**) Bar plots showing the change in intracellular pH peak amplitude. **** *p* < 0.0001, compared to the controls, using the two-tailed Student’s *t*-test and Mann–Whitney Rank Sum test (for MES-like cells). The number of cells/coverslips/cultures used in the experiments is indicated in the respective columns.

Expression of NBCe1 transcript and protein, as well as its potential biological significance in tumour development and growth have so far been investigated in epithelial tumours and cell lines, and only limitedly in glioblastoma cell lines [2]. Here, we investigated whether NBCe1 is expressed in the glioblastoma cellular subtypes, as classified by Neftel et al. [10] based on their expression profiles in MES-like hypoxia dependent, MES-like hypoxia *in*dependent, and AC-like GBM cells. First, NBCe1 protein abundance was determined by immunoblot analysis. Using an antibody raised against NBCe1, strong immunoreactive bands at ~110 kDa and ~130 kDa were detected in cell homogenates of all GBM cellular states, as previously described for mouse hippocampal and cortical astrocytes [14,15,16], (Figure 1A). These results were further confirmed by immunofluorescence, as shown in Figure 1B,D. NBCe1 revealed intracellular distribution (asterisks) in all GBM cells and additionally membrane localisation (arrows).

Moreover, to investigate whether NBC transport is active in GBM cells, intracellular pH recordings were performed in cultured cells loaded with the H^+^-selective dye BCECF (Figure 1E). When the bathing solution was switched from a HEPES-buffered, nominally CO_2_/HCO_3_^−^-free saline solution to saline buffered by 5% CO_2_/26 mM HCO_3_^−^, a transient intracellular acidification was observed in all glioblastoma cellular subtypes investigated. The peak amplitude of this acidification was different in the absence or presence of 30 µM of the NBC blocker S0859. Quantification of the data (Figure 1F) showed that the peak amplitude upon inhibition of NBC was significantly larger in MES-like hypoxia dependent (0.43 ± 0.01 pH units), MES-like hypoxia *in*dependent (0.39 ± 0.01 pH units) and AC-like GBM cells (0.34 ± 0.01 pH units), compared to their controls in the absence of the inhibitor (0.30 ± 0.01, 0.32 ± 0.01 and 0.26 ± 0.00 pH units, respectively; **** *p* < 0.0001, using two-tailed unpaired Student’s *t*-test (in AC-like cells) and the Mann–Whitney Rank Sum test (in both MES-like cells; Figure 1F). These data demonstrate the presence of active NBC transport in all glioblastoma cellular subtypes investigated.

### 2.2. Induction of Chemical Hypoxia Differentially Regulates NBCe1 Protein Abundance and Activity in GBM Cellular States

**Figure 2:**
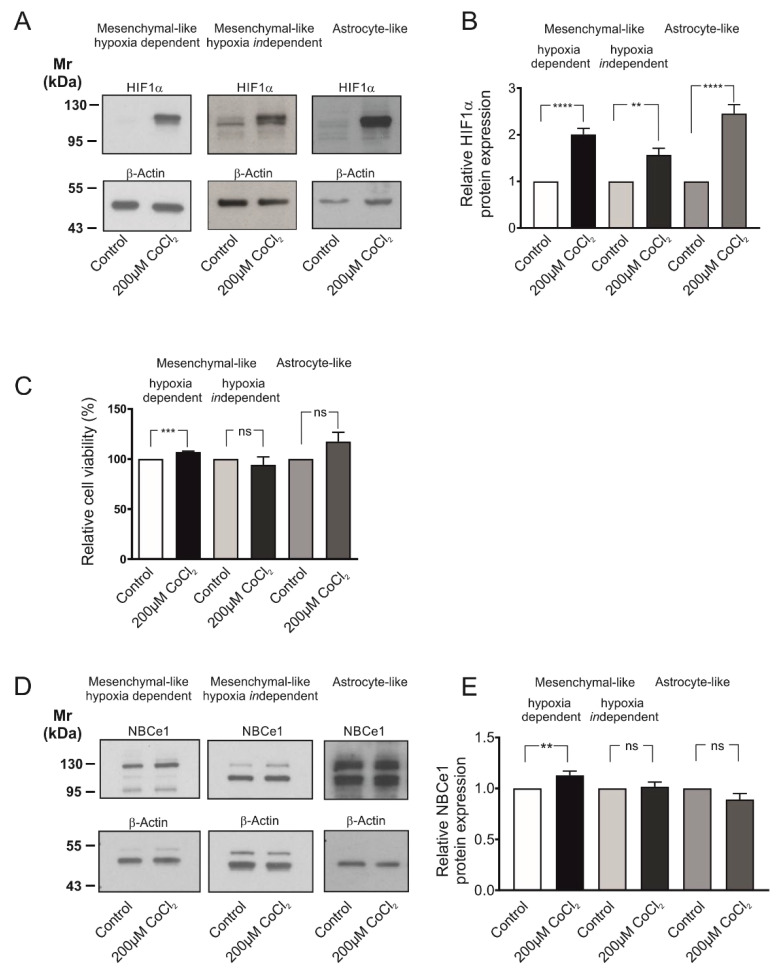
Cell viability and regulation of NBCe1 protein abundance in different glioblastoma cellular subtypes following exposure to chemical hypoxia. (**A**) Immunoblot analysis for hypoxia-inducing factor 1 α (HIF-1α) in MES-like hypoxia-dependent and hypoxia-*in*dependent cells, as well as in astrocyte-like glioblastoma cell types following exposure of the cells to 200 µM CoCl_2_ for 24 h. (**B**) Quantification of the data. ** *p* < 0.01 and **** *p* < 0.0001 for significant increase after densitometric analysis of the signal ratio HIF-1α: β-Actin and two-tailed unpaired Student’s *t*-test. Data are presented as mean ± S.E.M. The blots are representative for 5–9 different experiments; 10–30 µg protein was loaded per lane. The value of untreated controls was set to 1. (**C**) Viability of GBM cells following chemical hypoxia was quantified with the MTT assay. Data are given as relative numbers (%) following 200 µM CoCl_2_, compared to the untreated controls (*** *p* < 0.001, and ns: not significant using two-tailed unpaired Student’s *t*-test, *n* = 3). (**D**) Immunoblot analysis of NBCe1 protein in MES-like hypoxia-dependent and hypoxia-*in*dependent cells, as well as in astrocyte-like GBM cells following induction of chemical hypoxia for 24 h. (**E**) Quantification of the data. ** *p* < 0.01 for significant increase and ns: not significant after densitometric analysis of the signal ratio NBCe1: β-Actin and two-tailed unpaired Student’s *t*-test. Data are presented as mean ± S.E.M. The blots are representative for 3–10 different experiments. 10–30 µg protein was loaded per lane. The value of untreated controls was set to 1.

Having shown that GBM cells express active NBC transport, we next asked whether NBCe1 is regulated under hypoxic conditions. To that end, the model of chemical-induced hypoxia was used [17]. First, we elucidated whether treatment of the different glioblastoma cellular subtypes with 200 µM CoCl_2_ was able to stabilize hypoxia-inducible factor 1α (HIF-1α) levels in the cells, thus mimicking hypoxic conditions. Protein abundance of HIF-1α was determined by western blot analysis. As shown in Figure 2A, HIF-1α protein expression was low or absent in whole cell homogenates of controls. Following treatment of the cells with 200 µM CoCl_2_ for 24 h, a strong immunoreactive band ~110–120 kDa, corresponding to full length HIF-1α protein, was detected. Intensity of the band was significantly increased in CoCl_2_-treated cells (2.00 ± 0.13-fold, 1.57 ± 0.14-fold and 2.45 ± 0.19-fold) for MES-like hypoxia dependent, MES-like hypoxia *in*dependent and AC-like GBM cells (Figure 2B), compared to the untreated controls (** *p* < 0.01 and **** *p* < 0.0001, using the two-tailed unpaired Student’s *t*-test, *n* = 5–9), thus demonstrating induction of hypoxia.

Induction of chemical hypoxia did not compromise cell survival, as tested by MTT assay and shown in Figure 2C. Following treatment with CoCl_2_ for 24 h, a cell viability of 107.20 ± 0.75%, 94.13 ± 8.00% and 117.40 ± 9.25% for MES-like hypoxia-dependent, MES-like hypoxia-*in*dependent and AC-like GBM cells respectively, was measured, showing that cell viability was significantly increased in MES-like hypoxia-dependent but not in MES-like hypoxia-*in*dependent or AC-like GBM cells, compared to the untreated controls (using the two-tailed unpaired Student’s *t*-test, *n* = 3).

Consequently, as there was no cytotoxic effect on the cells after 24 h treatment with 200 µM CoCl_2_, this hypoxia model was used for the subsequent experiments. Figure 2D illustrates NBCe1 protein abundance in the three glioblastoma cellular subtypes following exposure to 200µM CoCl_2_ for 24 h. In controls, two immunoreactive bands, at ~110 kDa and ~130 kDa were detected, as previously reported [18]. Following induction of chemical hypoxia, NBCe1 protein was significantly increased in MES-like hypoxia-dependent cells (1.13 ± 0.04-fold) but not in MES-like hypoxia-*in*dependent (1.02 ± 0.05-fold) or in AC-like GBM cells (0.89 ± 0.06-fold), compared to the untreated controls (** *p* < 0.01, using the two-tailed unpaired Student’s *t*-test, *n* = 3–10, Figure 2E).

**Figure 3:**
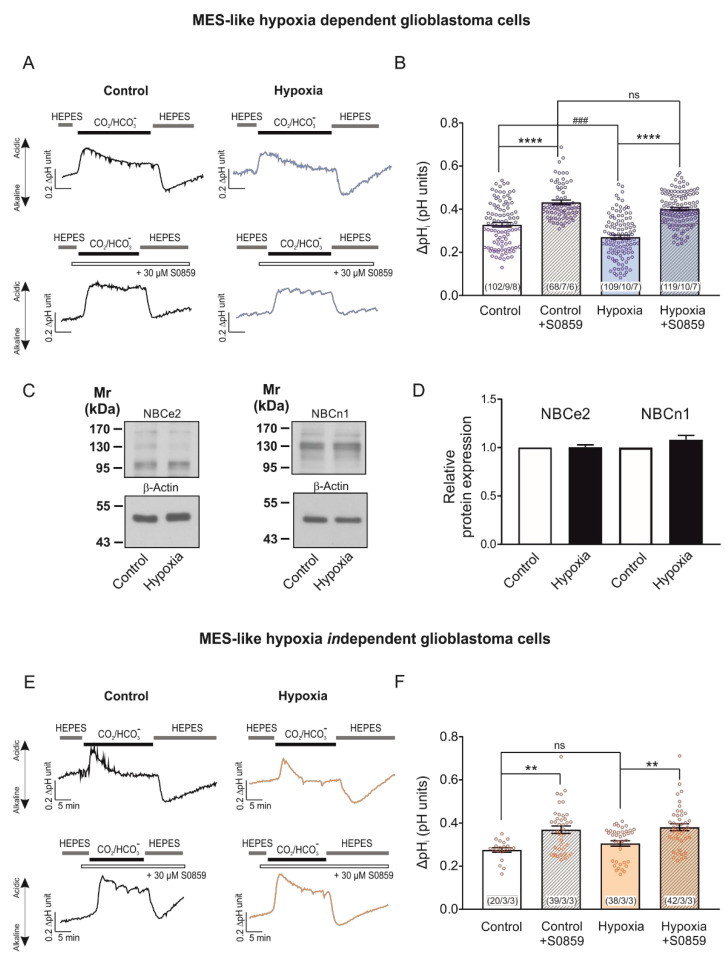
Regulation of NBCe1 transport activity in different glioblastoma cellular subtypes, following induction of chemical hypoxia for 24 h. Original recordings of intracellular pH in cultured hypoxia-dependent (**A**) and hypoxia-*in*dependent (**E**) mesenchymal-like glioblastoma cell types during change of perfusion solution from HEPES-buffered to saline solution buffered by 5% CO_2_/26 mM HCO_3_^-^ and back to HEPES and following exposure to 200 µM CoCl_2_ for 24 h the presence or absence of 30 µM of the NBC inhibitor S0859. (**C**) Immunoblot analysis of NBCe2 and NBCn1 protein in MES-like hypoxia-dependent GBM cells following chemical hypoxia for 24 h. (**D**) Quantification of the data. Not significant after densitometric analysis of the signal ratio NBCe2: β-actin and NBCn1: β-actin and two-tailed unpaired Student’s *t*-test. Data are presented as mean ± S.E.M. The blots are representative for six-seven different experiments; 20 µg protein was loaded per lane. The value of untreated controls was set to 1. (**B**,**F**) Bar plots showing the change of intracellular pH peak amplitude. ** *p* < 0.01 and **** *p* < 0.0001 for significant increase, ^###^ *p* < 0.001 for significant decrease; ns: not significant, using one-way ANOVA and Bonferroni post hoc test. The number of cells/coverslips/cultures used in the experiments is indicated in the respective columns.

To test whether the observed NBCe1 upregulation following chemical hypoxia resulted in increased NBC transport capacity in MES-like hypoxia-dependent GBM cells, we performed intracellular pH recordings. In MES-like hypoxia-dependent GBM cellular subtype, the CO_2_/HCO_3_^−^- induced intracellular acidification was significantly decreased in cells that had been exposed to chemical hypoxia (0.27 ± 0.01 pH units; Figure 3A,B), compared to the untreated controls (0.33 ± 0.01 pH units). This effect was prevented after application of 30 µM S0859 (0.40 ± 0.01 pH units and 0.43 ± 0.00 pH units for hypoxia and controls, respectively, Figure 3A,B). The latter result demonstrates that the decreased hypoxia-induced intracellular acidification between control and hypoxia-exposed cells can be attributed to increased activation of NBC transport (^###^ *p* < 0.001 for significant decrease and **** *p* < 0.0001 for significant increase using one-way ANOVA and Bonferroni post hoc test, *n* = 6–8; Figure 3B). To identify the molecular identity of the NBC responsible for increased NBC transport following hypoxia, we have performed immunoblot analysis and determined protein expression of NBCe2 (SLC4A5) and NBCn1 (SLC4A7) in control MES-like hypoxia-dependent GBM cells and following chemical hypoxia. The results are shown in Figure 3C,D. Both NBCe2 protein (~126 kDa) and NBCn1 (~136 kDa) expression was comparable in cells exposed to hypoxia (1.00 ± 0.02-fold and 1.08 ± 0.05-fold for NBCe2 and NBCn1, respectively), compared to the controls (not significant, using the two-tailed unpaired Student’s *t*-test, *n* = 6–7). These results indicate that the hypoxia-induced upregulation of NBC transport can be attributed to the action of NBCe1.

We have previously shown that post-translational modifications of NBCe1, such as phosphorylation at distinct serine residues is a potent regulatory mechanism for NBCe1 transport capacity that is not accompanied by changes on protein levels [14,16]. Therefore, as a next step we investigated whether chemical hypoxia for 24 h may regulate NBCe1 transport activity, regardless of regulation of NBCe1 protein abundance, as is the case in MES-like *in*dependent GBM cells. In an MES-like hypoxia-*in*dependent GBM cellular state, no significant differences could be observed in pH_i_ peak amplitudes (Figure 3E) after induction of chemical hypoxia for 24 h (0.31 ± 0.01 pH units), compared to the untreated controls (0.28 ± 0.01 pH units). Notably, NBC transport was active at both groups, as indicated by the increase of peak amplitude in the presence of S0859 (0.38 ± 0.01 pH units and 0.37 ± 0.02 pH units for hypoxia and controls, respectively; ** *p* < 0.01, using one-way ANOVA and Bonferroni post hoc test, *n* = 3, Figure 3F). 

Taken together, these results demonstrate that hypoxia has a different effect on NBCe1 protein and activity in MES-like hypoxia-dependent and hypoxia-*in*dependent GBM cells.

### 2.3. Chemical Hypoxia Does Not Alter Secretion of Cytokines by MES-like Hypoxia-Dependent Glioblastoma Cells

**Figure 4:**
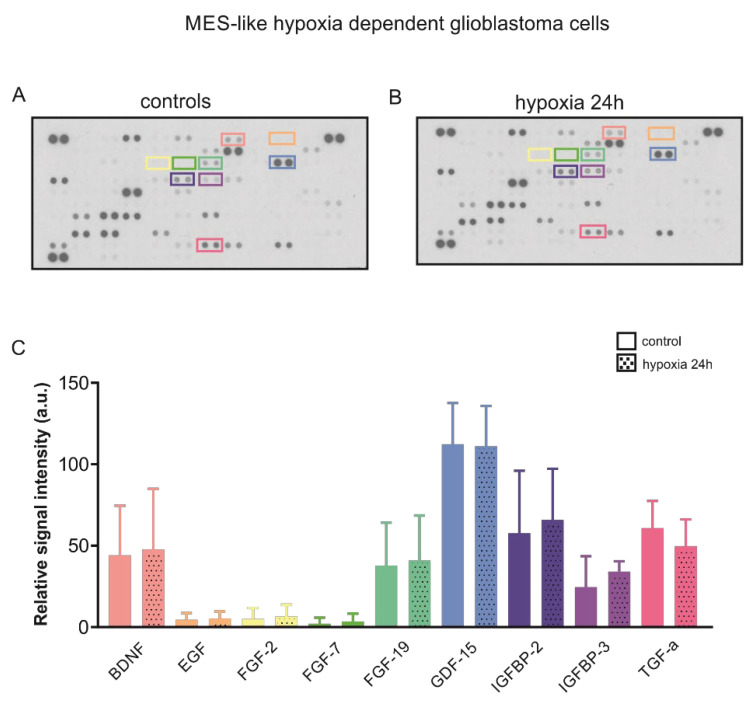
Cytokine profile following hypoxia in MES-like hypoxia-dependent GBM cells. Secretome analysis in supernatant from control mesenchymal-like hypoxia dependent glioblastoma cells (**A**) and in those exposed to chemical hypoxia for 24 h (**B**) using the Proteome profiler^TM^ Human XL Cytokine Array (R&D Systems). (**C**) Quantification of the data. No differences were observed in the relative levels of selected secreted cytokines (coloured boxes).

The crosstalk between HIF and cytokines in cancer is well documented [19]. We therefore asked whether the secretion of relevant growth factors by MES-like hypoxia dependent glioblastoma cells is different following chemical hypoxia for 24 h. Therefore, we determined the relative levels of secreted cytokines in the supernatant of cultured cells, as described in Materials and Methods. The results are presented in Figure 4A and the quantification in Figure 4B. Out of the 105 soluble human secreted proteins spotted on the membranes, we have selected nine growth factors for further analysis. Epidermal growth factor (EGF), and Fibroblast growth factor (FGF)-2 and FGF-7 were not detectable. No differences on the relative amount of protein of the growth factors (coloured boxes) brain derived neurotrophic factor (BDNF), FGF-19, growth differentiation factor (GDF)-15, insulin-like growth factor-binding protein (IGFBP)-2, IGFBP-3, and transforming growth factor (TGF)-α were detected between the experimental groups. These data indicate that with regard to these cytokines, induction of chemical hypoxia did not alter the microenvironment of MES-like hypoxia-dependent GBM cells.

### 2.4. Involvement of TGF-β Pathway in Hypoxia-Induced Regulation of NBC Transport in MES-Like Hypoxia Dependent GBM Cells

**Figure 5:**
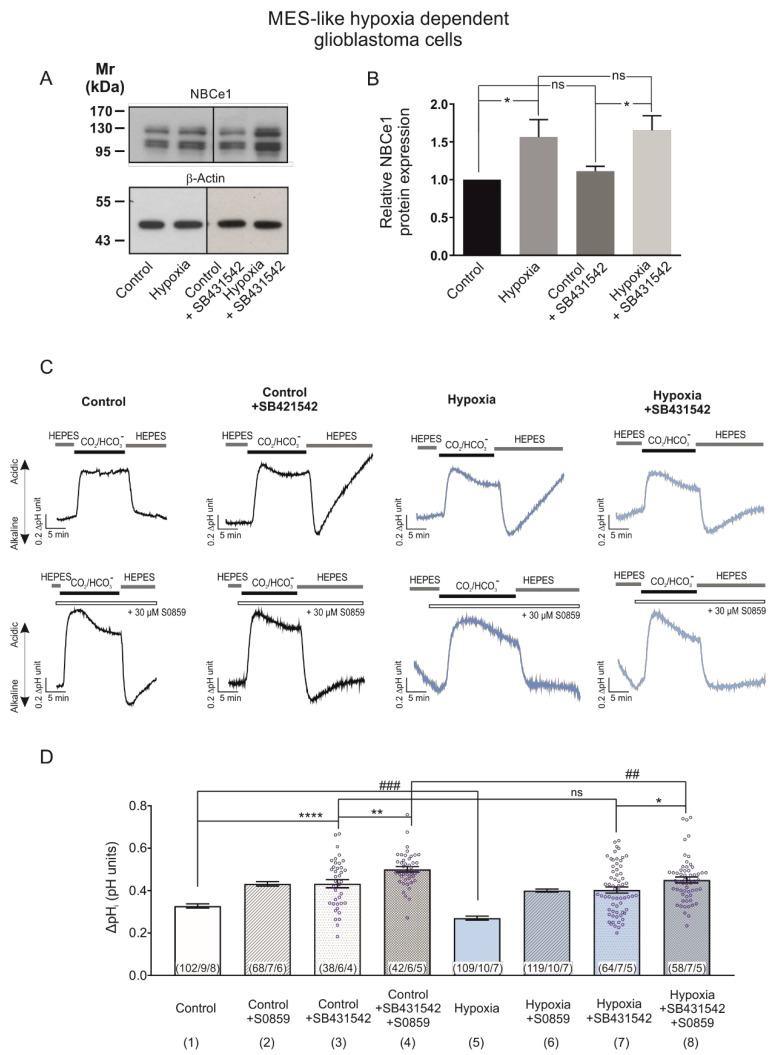
TGF-β pathway is involved in hypoxia-induced NBCe1 regulation in MES-like hypoxia-dependent GBM cells. (**A**,**B**) Immunoblot analysis for NBCe1 in controls and following exposure to chemical hypoxia in MES-like hypoxia-dependent GBM cells in the presence of 10 µM SB431542, an inhibitor of TGF-β type I receptor (ALK5) in addition to ALK4, and ALK7. * *p* < 0.05 for significant increase after densitometric analysis of the signal ratio NBCe1: β-Actin and one-way ANOVA and Bonferroni post hoc test. Data are presented as mean ± S.E.M. ns: not significant. The blots are representative for four-five different experiments; 30 µg protein was loaded per lane. The value of untreated controls was set to 1. (**C**) Determination of NBCe1 transport. Original recordings of intracellular pH in cultured MES-like hypoxia-dependent glioblastoma cells during change of perfusion solution from HEPES-buffered to saline solution buffered by 5% CO_2_/26 mM HCO_3_^-^ and back to HEPES and following exposure to 200 µM CoCl_2_ for 24 h in the presence or absence of 30 µM of the NBC inhibitor S0859 and of 10 µM SB431542. (**D**) Bar plots showing the change of intracellular pH peak amplitude. * *p* < 0.05, ** *p* < 0.01, **** *p* < 0.0001, for significant increase and ^##^ *p* < 0.01, ^###^ *p* < 0.001 for significant decrease; ns: not significant. The data in columns 1, 2, 5 and 6 are the same as in Figure 3B, columns 1, 2, 3 and 4, respectively. The number of cells/coverslips/cultures used in the experiments is indicated in the respective columns.

We have previously shown that NBCe1 is a target of the TGF-β pathway in cortical astrocytes [18]. Moreover, increased activation of TGF-β pathway was observed in glioblastoma, concomitant with TGF-β-based maintenance of tumour stem cell characteristics [20,21]. In epithelial cancers, TGF-β may even act synergistically with hypoxia [22]. With this background in mind, we investigated NBCe1 protein abundance and NBCe1 transport activity in MES-like hypoxia-dependent GBM cells using 10 µM SB431542, an inhibitor of TGF-β type I receptor (ALK5) in addition to ALK4, and ALK7 [23]. As shown in Figure 5A, immunoblot analysis revealed comparable amounts of NBCe1 protein in untreated MES-like hypoxia-dependent GBM cells in the presence (1.11 ± 0.06-fold, lane 3) or absence of SB431542 (Figure 5A, lane 1). Following induction of chemical hypoxia in the presence of SB431542, NBCe1 protein abundance was significantly upregulated (1.65 ± 0.19-fold) (* *p* < 0.05, using one-way ANOVA and Bonferroni post hoc test, *n* = 4–5), but was not significantly different compared to hypoxia alone (1.56 ± 0.23-fold). 

Subsequently, since TGF-β exerts its actions through several signalling pathways able to post-translationally regulate NBCe1 and thereby indirectly influence NBCe1 transport, we determined NBC activity in the presence or absence of SB431542.

Representative traces from pH recordings are shown in Figure 5C. As shown in Figure 5D, inhibition of TGF-β signalling with SB431542 significantly increased intracellular acidification (column 3; 0.43 ± 0.02 pH units), compared to the untreated controls (column 1; 0.33 ± 0.01 pH units). In the presence of TGF-β inhibitor, however, no difference in the intracellular acidification was observed between controls (column 3; 0.43 ± 0.02 pH units) and cells exposed to chemical hypoxia (column 7; 0.40 ± 0.01 pH units), while hypoxia alone significantly decreased intracellular acidification (column 1 vs. 5; 0.33 ± 0.01 pH units and 0.27 ± 0.01 pH units), compared to the controls. 

In addition, the intracellular acidification following hypoxia in the presence of inhibitors of TGF-β signalling and of NBC was significantly decreased (column 8; 0.45 ± 0.01 pH units) compared to the respective controls (column 4, 0.50 ± 0.01 pH units). These results suggest that the hypoxia-induced upregulation of NBCe1 is at least partly mediated by the TGF-β pathway (* *p* < 0.05, ** *p* < 0.01, **** *p* < 0.0001 for significant increase and ^##^ *p* < 0.01 and ^###^ *p* < 0.001 for significant decrease).

### 2.5. Hypoxia-Induced Regulation of HIF-1α and NBCe1 Transport Is Prevented by Low Extracellular pH in MES-Like Hypoxia Dependent GBM Cells

Adaptation to pH changes due to hypoxia is essential for tumour growth and survival and bicarbonate buffering is important for this adaption process [2,12,24,25]. Moreover, in Ls174T (colon cancer) cells, hypoxic induction of *Slc4a4* is regulated by HIF-1α and sequences for HIF-1α binding sites were found in the *Slc4a4* promoter [12]. On the other side, lactic acidosis for 24 h or extracellular acidosis for 48 h represses the hypoxia response by the inhibition of HIF-1α synthesis in breast cancer cells [26,27].

**Figure 6:**
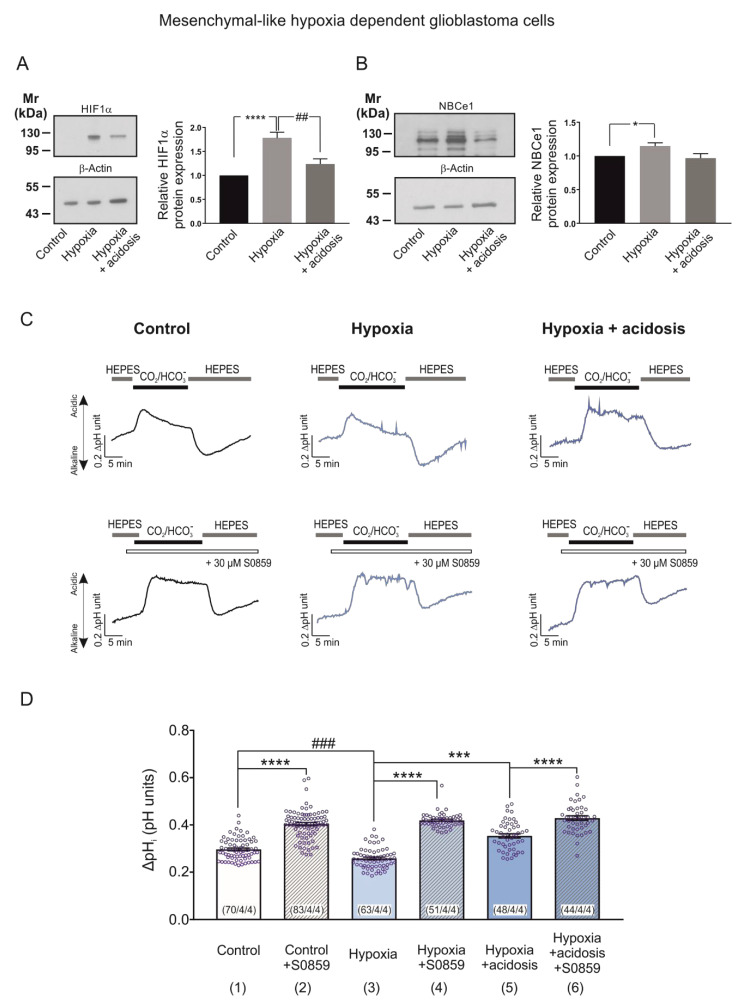
Hypoxia-induced NBCe1 regulation in MES-like hypoxia dependent GBM cells is prevented by low extracellular pH. (**A**,**B**) Immunoblot analysis for HIF-1α (**A**) and NBCe1 (**B**) in MES-like hypoxia-dependent GBM cells following chemical hypoxia and combined with extracellular acidosis (pH 6.8) for 24 h. * *p* < 0.05, **** *p* < 0.0001 for significant increase and ^##^ *p* < 0.01 for significant decrease after densitometric analysis of the signal ratio HIF-1α: β-actin and NBCe1: β-actin using one-way ANOVA and Bonferroni post-hoc test. Data are presented as mean ± S.E.M. The blots are representative for six different experiments; 10 µg protein was loaded per lane. The value of untreated controls was set to 1. (**C**) Original recordings of intracellular pH in cultured MES-like hypoxia dependent GBM cells during change of perfusion solution from HEPES-buffered to saline solution buffered by 5% CO_2_/26 mM HCO_3_^-^ and back to HEPES in controls and following exposure to 200 µM CoCl_2_ and acidosis for 24 h in the presence or absence of 30 µM of the NBC inhibitor S0859. (**D**) Bar plots showing the change in peak amplitude of acidification. *** *p* < 0.001, **** *p* < 0.0001 for significant increase and ^###^ *p* < 0.001 for significant decrease, using one-way ANOVA and Bonferroni post hoc test. The number of cells/coverslips/cultures used in the experiments is indicated in the respective columns.

We thus investigated the NBCe1-mediated response of MES-like hypoxia-dependent GBM cells during induction of chemical hypoxia in an acidic environment (extracellular pH 6.8). Figure 6A,B illustrate immunoblotting for HIF-1α and NBCe1 in MES-like hypoxia-dependent GBM cells following exposure to 24 h CoCl_2_ and extracellular acidosis. The hypoxia-induced HIF-1α upregulation (Figure 6A, lane 2) was prevented by low pH (1.78 ± 0.12-fold and 1.24 ± 0.10-fold for hypoxia and hypoxia & acidosis, respectively), compared to the control cells (**** *p* < 0.001 for significant increase compared to control and ^##^ *p* < 0.01 for significant decrease, compared to hypoxia, using one-way ANOVA and Bonferroni post hoc test, *n* = 6).

With regard to NBCe1 protein abundance, as shown in Figure 6B, although hypoxia-induced significant NBCe1 upregulation in MES-like hypoxia-dependent GBM cells (1.15 ± 0.05-fold; Figure 5B, lane 2) was decreased during chemical hypoxia in combination with extracellular pH of 6.8 (0.97 ± 0.07-fold; Figure 6B, lane 3) no significant differences were detected in hypoxia in the presence or absence of extracellular acidosis (Figure 6B, lanes 2 and 3). * *p* < 0.05 for significant increase, one-way ANOVA and Bonferroni post hoc test, *n* = 6).

To test the functional consequences of the dual challenge of hypoxia and extracellular acidosis, we performed intracellular pH recordings. In MES-like hypoxia-dependent GBM cells, the CO_2_/HCO_3_^-^-induced intracellular acidification was significantly decreased in cells that had been exposed to chemical hypoxia alone (column 3; 0.26 ± 0.00 pH units; Figure 6D). It was however significantly increased in cells exposed to chemical hypoxia and extracellular acidosis (column 5; 0.35 ± 0.01 pH units, Figure 6D), compared to the untreated controls (column 1; 0.30 ± 0.01 pH units). These effects were prevented after application of 30 µM S0859 (column 4; 0.42 ± 0.00 pH units, column 6; 0.43 ± 0.01 pH units and column 2; 0.40 ± 0.01 pH units for hypoxia alone, hypoxia with extracellular acidosis and controls, respectively). This indicates that the hypoxia-induced upregulation of NBCe1 transport is prevented in the presence of extracellular acidosis (*** *p* < 0.001, **** *p* < 0.0001 for significant increase and ^###^ *p* < 0.001 for significant decrease using one-way ANOVA and Bonferroni post hoc test, *n* = 3–4; Figure 6D).

### 2.6. Extracellular Acidosis Does Not Regulate Hypoxia-Induced HIF-1α Expression but Reduces NBCe1 Transport in MES-Like Hypoxia Independent GBM Cells

**Figure 7:**
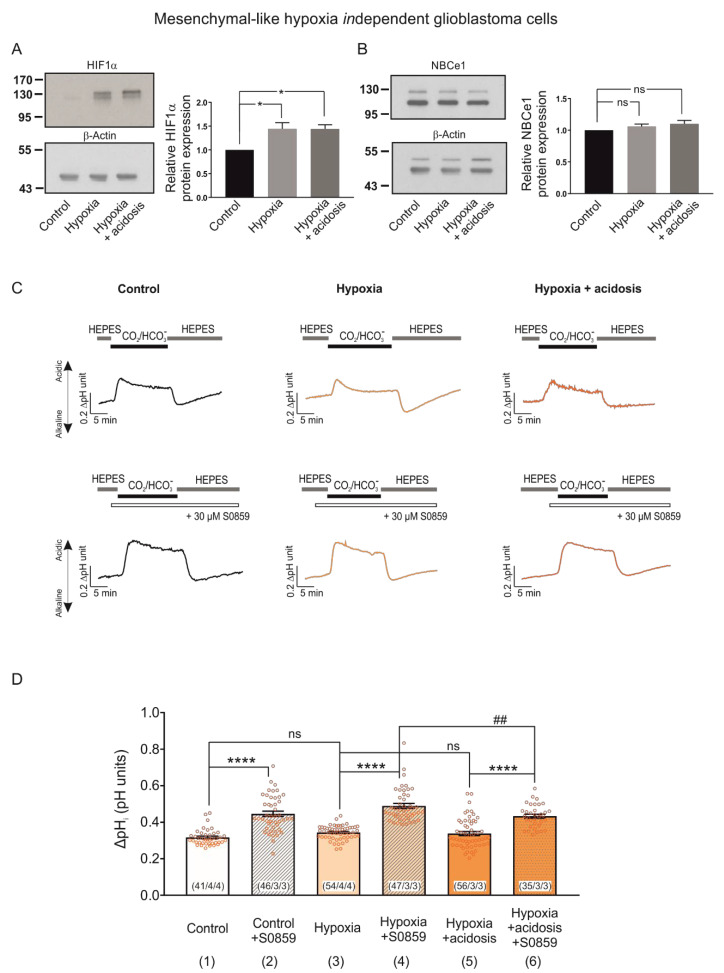
HIF-1α and NBCe1 regulation in MES-like hypoxia *in*dependent GBM cells during exposure to chemical hypoxia and extracellular acidosis. (**A**,**B**) Immunoblot analysis for HIF-1α (**A**) and NBCe1 (**B**) in MES-like hypoxia-*in*dependent GBM cells following chemical hypoxia combined with extracellular acidosis (pH 6.8) for 24 h. * *p* < 0.05 and ns: not significant after densitometric analysis of the signal ratio HIF-1α: β -actin and NBCe1: β actin and one-way ANOVA and Bonferroni post hoc test. Data are presented as mean ± S.E.M. The blots are representative for six different experiments; 10 µg protein was loaded per lane. The value of untreated controls was set to 1. (**C**) Original recordings of intracellular pH changes in cultured MES-like hypoxia-*in*dependent GBM cells during change of perfusion solution from HEPES to CO_2_/HCO_3_^-^ and back to HEPES in controls and following exposure to 200 µM CoCl_2_ and extracellular acidosis for 24 h in the presence or absence of 30 µM of the NBC inhibitor S0859. (**D**) Bar plots showing the changes in the peak amplitude. ^##^ *p* < 0.01 for significant decrease and **** *p* < 0.0001, for significant increase and ns: not significant using one-way ANOVA and Bonferroni post hoc test. The number of cells/coverslips/cultures used in the experiments is indicated in the respective columns.

To determine the effect of extracellular acidosis combined with hypoxia on NBCe1 in MES-like hypoxia-*in*dependent GBM cells, we used the same experimental design as described before (Figure 6). Figure 7A,B illustrate immunoblotting for HIF-1α and NBCe1 in MES-like hypoxia-*in*dependent cells following exposure to 24 h CoCl_2_ and accompanied by extracellular acidosis (pH of 6.8).

In contrast to the results obtained in MES-like hypoxia-dependent GBM cells (Figure 6A), the hypoxia-induced HIF-1α upregulation persisted in the presence of low extracellular pH (Figure 7A, 1.44 ± 0.13-fold and 1.44 ± 0.09-fold for hypoxia and hypoxia & acidosis, respectively, compared to the controls; * *p* < 0.05, using one-way ANOVA and Bonferroni post hoc test, *n* = 6). Moreover, protein level of NBCe1 (Figure 7B) was comparable between the experimental groups (1.06 ± 0.04-fold and 1.10 ± 0.05-fold for hypoxia and hypoxia in combination with acidosis, respectively, compared to the untreated controls, not significant (ns) using one-way ANOVA and Bonferroni post hoc test, *n* = 6).

In contrast, NBCe1 transport was less activated following chemical hypoxia combined with extracellular acidosis, compared to hypoxia alone. In MES-like hypoxia-independent GBM cells CO_2_/HCO_3_^−^ -induced pH changes were similar between the experimental groups (i.e., controls, column 1; 0.32 ± 0.01 pH units, cells exposed to hypoxia alone, column 3; 0.34 ± 0.01 pH units, and during extracellular acidosis, column 5; 0.34 ± 0.01 pH units). In the presence of the NBC inhibitor S0859, in contrast, the peak amplitude of the pH change was reduced in hypoxia in combination with acidosis (column 6; 0.43 ± 0.01 pH units), compared to hypoxia alone (column 4; 0.49 ± 0.01 pH units), indicating reduced activation of NBC (**** *p* < 0.0001 for significant increase and ^##^ *p* < 0.01 for significant decrease using one-way ANOVA and Bonferroni post hoc test, *n* = 3–4).

Taken together, these results suggest that the dual challenge of hypoxia and extracellular acidosis triggers distinct NBCe1-mediated adaptation mechanisms in the two meta-modules of MES-like GBM cells.

## 3. Discussion

Dysregulation of ion balance in tumours and their environment has emerged as a key factor affecting tumour formation, survival, and relapse. In order to establish and sustain the reversed pH gradients between the intracellular and extracellular compartment, compared to non-cancer cells, acid-base transporters should be regulated accordingly in tumour cells. Based on these considerations, pH_i_ regulatory proteins, especially acid extruders such as the V-ATPase and the Na^+^/H^+^ exchanger (NHE1), have been a focus of cancer research [2]. In the context of glioblastoma, the relative expression of certain pH regulatory proteins, such as the subunit G1 of the V-ATPase is even considered as a prognostic marker [28].

While the impact of NBCs on cancer biology is appreciated, current knowledge of their action and regulation derives mainly from epithelial cancers [1] and their role on GBM is far from being understood. Since, in GBM, transcriptionally different malignant cells that dynamically populate each tumour have been identified [10], the adaption of the results from the largely homogenous epithelial tumours to glioblastoma is limited. Moreover, the few available studies on NBC in GBM cannot be directly compared to each other due to differences between experimental settings and culture models used among them such as monolayers vs. 3D spheroids, likely glioblastoma (U87) and disease glioblastoma (T98G) cell lines [12], primary cultures, and quiescent or non-glioblastoma stem cells [13].

In our study, we take advantage of the classification of malignant cells in glioblastoma into four main cellular subtypes and six meta-modules according to Neftel et al. [10] and we focus on the regulation of the key acid extruder NBCe1 protein and its activity. We tested the hypothesis that intratumoral cellular heterogeneity is accompanied by functional heterogeneity with regard to pH regulatory proteins, reflected as a distinct response to the tumour microenvironment. To that end, MES-like hypoxia-dependent and hypoxia-*in*dependent cells, as well as AC-like glioblastoma cells were used and their response to chemical hypoxia alone or in combination with extracellular acidosis was investigated. Considering that the cell states described by Neftel et al. [10] have a high degree of plasticity and are likely to shift according to the microenvironment into NPC-like or OPC-like states, recent work has shown that, in cell culture conditions, predominantly the MES- and AC-like states are maintained [11]. Although it is expected that hypoxia treatment of the cells results in a transcriptional shift towards the hypoxia-dependent state, the respective adaptation is found to be heterogeneous.

Our results show that all glioblastoma cellular subtypes investigated express NBCe1 protein, as determined by the use of an antibody specific against NBCe1 [14], as well as NBC activity, reflected by the increased intracellular acidification following inhibition of NBC (Figure 1). During exposure to CO_2_/HCO_3_^-^, the free diffusion of CO_2_ into the cell and the subsequent conversion to H^+^ and HCO_3_^-^ induces an acute intracellular acid load. Activation of acid-extruding NBCs regulates [H^+^]*_i_* from the CO_2_-induced acid load. Consequently, inhibition of NBC activity leads to increased intracellular acidification [29]. We also show that following induction of chemical hypoxia, NBC activity is differentially regulated in the transcriptionally distinct glioblastoma cells: hypoxia significantly upregulated NBCe1 protein abundance, but not protein levels of NBCe2 and NBCn1 and enhanced NBCe1 transport in MES-like hypoxia-dependent cells, but not in hypoxia-*in*dependent or AC-like cells (Figure 2, Figure 3 and Figure 4). These results contrast with the observation in U87 and T98G cell lines where hypoxia had no effect on *Nbce1* expression, whereas in spheroids (in normoxia) from U87, inhibition of NBC increased acidification of pH_i_ [12]. In the epithelial Ls174T, hypoxia upregulated *Scl4a4* [12], while, in monolayers of several epithelial cancer cell lines, bicarbonate transport was characterized as being hypoxia insensitive [30].

Besides hypoxia, lactic acidosis is one of the main microenvironmental hallmarks of solid tumours. However, the consequence of a putative hypoxia–acidosis interaction has been controversially discussed [31]. Our results in MES-like hypoxia-dependent GBM cells show that both the hypoxia-induced HIF-1α expression (Figure 6A) and the upregulation of NBCe1 activity (Figure 6D) were prevented in the presence of acidosis, suggesting NBCe1 regulation in an HIF-1α-dependent manner. Along this line, in Ls174T cells, knocking down of HIF-1α diminished the hypoxia-dependent *Scl4a4* upregulation and sequences for HIF binding sites were found in the *Slc4a4* promoter, indicating that hypoxia might be a transcriptional regulator of *Nbce1* [12]. In contrast, in MES-like hypoxia-*in*dependent GBM cells, when hypoxia was accompanied by extracellular acidosis, NBCe1 transport was less activated, compared to hypoxia alone (Figure 7D), without changes in NBCe1 protein abundance in an HIF-1α-independent manner. Taking into consideration that acidic pH and tumour hypoxia negatively regulate mTORC1 [32] together with the observation that mTOR-mediated phosphorylation of NBCe1 at S^255–257^ enhances NBCe1 activity [14], the acidosis-induced decreased NBC activity might be attributed to post-translational modifications of NBCe1. It is also conceivable that extracellular acidosis and/or hypoxia may regulate trafficking of several NBCs to the membrane, resulting in altered surface NBCs and subsequently NBC activity.

Taken together, these data suggest that NBCe1 may operate as “hypoxia-response-gene” in a cell-type specific manner depending on the transcriptional signature.

What could be the biological significance of these data? Experimental evidence suggest that epithelial cancer cells use an NBC mechanism for HCO_3_^-^ import and regulation of intracellular pH, with varying molecular identities of NBC [1]. In case our results are exclusively attributed to NBCe1 activity, an assumption supported by the lack of upregulation of NBCe2 and NBCn1 following hypoxia (Figure 3C,D), it is important to note that NBCe1 may act as an acid loader or an acid extruder depending on the exact conditions. As an example, in astrocytes, NBCe1 operates with a stoichiometry 1Na^+^: 2HCO_3_^-^ and may act as an acid loader or an acid extruder depending on the membrane potential, pH, [HCO_3_^-^] and [Na^+^] [29,33]. In case the transporter operates in an inward fashion, and neglecting that other acid–base transporters could also be regulated as well, increased NBCe1 activity will contribute to the establishment and/or maintenance of an alkaline pH_i_, a cellular state that in epithelial tumours may promote cell proliferation, migration or invasion [34].

Depending on the intratumoral spatial distribution of each individual cell and the distance from blood vessels, cells with distinct transcriptional profiles are exposed to an altered microenvironment with several molecular variables. First, cells are exposed to non-overlapping gradients of protons and oxygen within the tumour. Based on our data we can speculate that regulation of NBCe1 may be different in both cases, not only in cells exhibiting distinct transcriptional profiles but also in the same cell subtype depending on its intratumoral spatial distribution. Second, alterations of the tumour microenvironment may occur due to varying compositions of pro- and anti-inflammatory cytokines. Among the plethora of relevant proteins, increased activity of TGF-β signalling is associated with poor prognosis in glioblastoma patients [35] and consequently, TGF-β has been considered as a promising therapeutic target. Our results show comparable growth factor profiles between controls and hypoxia-exposed MES-like hypoxia-dependent GBM cells, but TGF-β was not included in the membranes used (Figure 4). We have therefore inhibited TGF-β signalling using the ALK4/5/7 inhibitor SB431542 (Figure 5) and showed that hypoxia-induced enhanced NBC activation in MES-like hypoxia-dependent GBM cells is at least partly regulated by TGF-β signalling. To our knowledge, this is the first demonstration for a hypoxia-induced and TGF-β -mediated cell-type-dependent regulation of an acid-base transporter in GBM. TGF-β may act context-dependently as tumour suppressive or tumour promoting. Hypoxia induces TGF-β production [36,37], and even under normoxia, TGF-β can increase HIF-1α levels and enhance its binding capacity [38]. In glioblastoma, TGF-β is upregulated [21], promotes oncogenesis by maintaining the self-renewing capacity of glioblastoma cells [20,39], and induces angiogenesis [40]. We have previously shown that TGF-β signalling directly upregulates NBCe1 gene, protein and functional expression in cortical astrocytes [18]. Such a mode of regulation apparently does not apply in MES-like hypoxia-dependentGBM cells, since NBCe1 protein abundance was comparable under chemical hypoxia with or without inhibition of TGF-β receptor (Figure 5A). Thus, the mechanism underlying the hypoxia-induced and TGF-β -mediated enhanced NBCe1 activity (Figure 5C) may be attributed to either the increased surface abundance of NBCe1 and/or to altered NBCe1 phosphorylation via non-canonical signalling. Recently, it has been demonstrated that secretion of TGF-β by tumour-associated reactive astrocytes from GBM with a transcriptional phenotype linked to the JAK/STAT pathway may modulate the tumour microenvironment to become immunosuppressive [41]. In the same work, inhibition of the JAK/STAT pathway has been shown to shift the tumour microenvironment to become pro-inflammatory and thus more favourable for immune therapeutic approaches. We have previously shown regulation of NBCe1 by STAT3 in reactive astrocytes [15].

We are aware of the limitation of our findings in the present study due to its in vitro nature. Nevertheless, it is intriguing that molecular determinants (TGF-β, mTOR and STAT3) either in the glioblastoma cellular states and/or in the tumour microenvironment converge as regulators of NBCe1 activity. We therefore consider the results as the basis and starting point for further studies to uncover the molecular and functional network between key players, fundamental for identifying targets for new therapeutic approaches for GBM. At the same time, the modes of regulation uncovered in the present study highlight the need for functional studies to complement data derived from gene expression studies.

## 4. Materials and Methods

### 4.1. Antibodies and Reagents/Chemicals

The following antibodies were used as primary antibodies: rabbit polyclonal anti-SLC4A4 (NBCe1; Alomone, Jerusalem, Israel, Cat# ANT-075, RRID:AB_2341019 and Abcam, Cambridge, UK, Cat# ab78326, #ab38686 and Cat# ab30323, RRID:AB_777961) for western blots and immunocytochemistry, respectively; rabbit polyclonal anti-SLC4A5 (NBCe2; Proteintech, Planegg-Martinsried, Germany, Cat# 26150-1-AP, RRID:AB_2918099), rabbit polyclonal anti-SLC4A7 (NBCn1; Abcam, Cambridge, UK, Cat# ab82335, RRID:AB_10672662), mouse monoclonal anti-HIF-1α (Proteintech, Planegg-Martinsried, Germany, Cat# 20960-1-AP, RRID:AB_10732601), and anti-β-Actin (Developmental Studies Hybridoma Bank, Iowa City, USA Cat# jla20, RRID:AB_528068. The following antibodies were used as secondary antibodies: for immunofluorescence, goat anti-rabbit IgG coupled to AlexaFluor594 (Jackson ImmunoResearch Labs, West Grove, PA, USA, Cat#711-585-152, RRID:AB_2340621), for western blots, goat-anti-mouse or anti-rabbit IgG coupled to horseradish peroxidase (Jackson ImmunoResearch Labs, West Grove, PA, USA, Cat#715-475-151, RRID:AB_2340840 or Thermo Fisher Scientific, Waltham, MA, USA Cat#A10042, RRID:AB_2534017).

### 4.2. Cell Culture

Three cell lines were established and classified using bulk RNA-seq according to the transcriptional classification [10,11,42]. The GBM cells are distinguished based on their gene expression profiles. Two cell lines were classified as mesenchymal-like and were subdivided in a hypoxia-dependent (BTSC#1, former glioblastoma stem cells GSC) and a hypoxia-*in*dependent meta-module (BTSC#233). Another cell line was classified as astrocyte-like (BTSC#168) [42]. The cells from the three different cell lines were cultured as adherent monolayers in Minimum Essential Medium (MEM) supplemented with 10% Fetal Bovine Serum (FBS) and 1% Penicillin-Streptomycin-Neomycin (PSN) in either 25 cm² flasks or in coverslips. In order to induce chemical hypoxia, cells were treated with 200 µM cobalt chloride (CoCl₂) for 24 h, an agent that leads to the stabilization of HIF-1α and HIF-2α for 12–48 h and thus mimics hypoxia in normoxic conditions [17]. For induction of acidosis additionally to chemical hypoxia, cells were cultured in Dulbecco’s Modified Eagle’s Medium (DMEM) and supplemented with either 26 mM or 6.1 mM NaHCO_3_^-^ to result in an extracellular pH of 7.4 (controls) or 6.8 (acidosis), respectively, and exposed to 200 µM CoCl_2_. For pharmacological manipulation, cells were cultured in the presence or absence of 10 µM of the ALK4/5/7 inhibitor SB431542 [23] and perfused with or without 30 µM of the NBC inhibitor S0859 [43]. Subsequently, cultures were processed for MTT (3-(4,5-dimethylthiazol-2-yl)-2,5-diphenyltetrazolium bromide) assay, immunoblotting, immunofluorescence, or intracellular H^+^ recordings.

### 4.3. Immunocytochemistry

Immunofluorescence of cultures was performed as previously described [14]. The primary antibody (NBCe1 1:200) was diluted in PBS. Cells were incubated with secondary antibody goat anti-rabbit IgG coupled to Alexa 594 (1:400) for 1 h at RT and viewed with a Leica TCS SP8 confocal microscope (Wetzlar, Germany).

### 4.4. Immunoblotting

GBM cells were harvested and homogenized, and protein concentration was determined by Thermo Scientific NanoDrop 2000 spectrophotometer. Electrophoresis and blotting procedures were performed as described [18]. Primary antibodies were diluted as follows: HIF-1α 1: 1000, NBCe1 1:2000, NBCe2 1: 1000, NBCn1 1:2000, and β-actin 1:30,000. Blots were developed in enhanced chemiluminescence reagents and signals were visualized on X-ray films. Subsequently, films were scanned and the signal ratio “protein of interest: β –actin”, was quantified by densitometry. Differences in signal ratio were tested for significance and results with levels of * *p* < 0.05 were considered significant.

### 4.5. MTT Assay

Mesenchymal-like hypoxia dependent and independent, as well as astrocyte-like GBM cells were treated with 200 µM CoCl_2_ for 24 h and subsequently incubated with 1 mg/mL 3-(4,5-dimethylthiazol-2-yl)-2,5-diphenyl-tetrazolium bromide, MTT (Cat# M6494, Thermo Fisher Scientific, Waltham, MA, USA), for 4 h at 37 °C in a 5 % CO_2_ incubator, according to the manufacturer’s protocol. MTT is reduced to a coloured compound, formazan, by mitochondria. Addition of DMSO solubilizes the formazan, and therefore colour intensity can be measured using a Vector microplate reader (PerkinElmer, Waltham, MA, USA) [44]. All treatments were performed in triplicate.

### 4.6. Intracellular pH Imaging in GBM Cellular Subtypes

To measure intracellular pH in different GBM cells, we performed wide-field imaging using an upright microscope (Nikon Eclipse TE 200 or FN-1) with a 20× or 40× water-immersion objective. Cells were loaded with the acetoxymethyl ester of a proton-sensitive dye, BCECF-AM, by incubating them with 2 μM BCECF-AM in HEPES-buffered saline for 20 min at room temperature. Cells were then mounted and superfused continuously with HEPES-buffered saline containing (in mM) NaCl 140, KCl 5, α-D-glucose 10, NaH_2_PO_4_ 0.5, HEPES 10, MgCl_2_ 1, and CaCl_2_ 2. Alternatively, the following CO_2_/HCO_3_^-^-buffered saline was used (in mM): NaCl 114, KCl 5, NaH_2_PO_4_ 0.5, α-D-glucose 10, NaHCO_3_ 26 (21 at 35 °C), MgCl_2_ 1, and CaCl_2_ 2.

The fluorescence emission intensity of BCECF when excited at 488 nm changes inversely with a change in [H^+^]_i_ whereas the fluorescence emission intensity at 458 nm excitation is largely pH insensitive. BCECF was thus excited consecutively at 488 nm (proton-sensitive wavelength) and 458 nm (close to isosbestic point), and the changes in fluorescence emission were monitored at >505 nm. Images were obtained every 5 s (0.2 Hz) and the fluorescence ratio F(458)/F(488) was calculated from selected regions of interest, representing cell bodies. The ratio was converted into pH by using the nigericin-based calibration technique [45]. Cells were superfused with calibration solutions, containing nigericin 10 μM, NaCl 15 mM, KCl 130 mM, HEPES 20 mM, MgCl_2_ 1 mM and CaCl_2_ 1 mM, at pH 6.0, 6.5, 7.0, or 7.5.

Data in the figures are presented as mean ± S.E.M. The number of cells/coverslips/cultures used in the experiments is indicated in the respective columns; thereby “cultures” represent the independent experiments with a given GBM line, “coverslips” represent the total number of coverslips from the sum of the independent experiments and “cells” represent the total number of cells in which intracellular pH has been measured.

### 4.7. Determination of Levels Ofsecreated Human Cytokines

The Proteome profiler^TM^ Human XL Cytokine Array (R&D Systems) was used to determine the relative levels of selected secreted cytokines in control mesenchymal-like hypoxia dependent glioblastoma cells and in those exposed to chemical hypoxia for 24 h. To this end, cell culture supernatants were collected and processed according to the manufacturer’s instructions. 

### 4.8. Statistical Analysis

Statistical tests were performed as indicated in the text. All tests were performed in GraphPad Prism, Version 7.04 for Windows. Data were tested for normal distribution using a Shapiro–Wilk test and subsequently assessed for homogeneity of variance. If the data passed both tests, further analyses were performed using the two-tailed unpaired Student’s *t*-test. Values are reported as mean ± S.E.M., unless otherwise indicated. For datasets with non-normal distributions, the Mann–Whitney Rank Sum Test was used. For comparisons between more than two groups, one-way ANOVA and the Bonferroni post hoc test were applied. For all statistical tests, *p* < 0.05 was considered statistically significant and *p*-values are indicated in the figures as follows: * *p* < 0.05, ** *p* < 0.01, *** *p* < 0.001, **** *p* < 0.0001, ^##^ *p* < 0.01, and ^###^ *p* < 0.001.

## Data Availability

All data generated or analysed during this study are included in the manuscript.

## Abbreviations

NBCe1	Electrogenic sodium bicarbonate cotransporter 1
NBCe2	Electrogenic sodium bicarbonate cotransporter 2
NBCn1	Electroneutral sodium bicarbonate cotransporter 1
SLC	Solute carrier
TGF-β	Transforming growth factor beta
GBM	Glioblastoma multiforme
MES	Mesenchymal
AC	Astrocyte
MTT	3-(4,5-dimethylthiazol-2-yl)-2,5-diphenyltetrazolium bromide
BCECF	2’,7’-Bis-(2-Carboxyethyl)-5-(and-6)-Carboxyfluorescein
HIF	Hypoxia inducible factor
pH_i_	Intracellular potential of hydrogen
NHE1	Sodium-hydrogen exchanger 1
AE	Anion exchanger
CA	Carbonic Anhydrase
MCT4	Monocarboxylate transporter 4
ALK	Activin receptor-like kinase
mTOR	Mammalian target of rapamycin
JAK/STAT	Janus kinases/signal transducer and activator of transcription proteins
